# The elimination of a selectable marker gene in the doubled haploid progeny of co-transformed barley plants

**DOI:** 10.1007/s11103-012-9988-9

**Published:** 2012-11-21

**Authors:** Eszter Kapusi, Götz Hensel, María-José Coronado, Sylvia Broeders, Cornelia Marthe, Ingrid Otto, Jochen Kumlehn

**Affiliations:** 1 Plant Reproductive Biology, Leibniz Institute of Plant Genetics and Crop Plant Research (IPK) Gatersleben, Corrensstr. 3, 06466 Gatersleben, Germany; 2 Department for Applied Genetics and Cell Biology, University of Natural Resources and Applied Life Sciences, Muthgasse 11, Vienna, Austria; 3 Confocal Microscopy Unit, Hospital Universitario Puerta de Hierro de Majadahonda, Manuel de Falla 1, 28222 Madrid, Spain; 4 EU, JRC, Institute for Reference Materials and Measurements, RM Unit, Retieseweg 111, 2440 Geel, Belgium

**Keywords:** *Agrobacterium*-mediated transformation, Selectable marker-free, T-DNA, Doubled haploid, Barley

## Abstract

Following the production of transgenic plants, the selectable marker gene(s) used in the process are redundant, and their retention may be undesirable. They can be removed by exploiting segregation among the progeny of co-transformants carrying both the selectable marker gene and the effector transgene. Here we show that the doubled haploid technology widely used in conventional barley breeding programmes represents a useful means of fixing a transgene, while simultaneously removing the unwanted selectable marker gene. Primary barley co-transformants involving *hpt::gfp* (the selectable marker) and *gus* (a model transgene of interest) were produced via *Agrobacterium*-mediated gene transfer to immature embryos using two respective T-DNAs. These plants were then subjected to embryogenic pollen culture to separate independently integrated transgenes in doubled haploid progeny. A comparison between 14 combinations, involving two *Agrobacterium* strains carrying various plasmids, revealed that the highest rate of independent co-transformation was achieved when a single *Agrobacterium* clone carried two binary vectors. Using this principle along with *Agrobacterium* strain LBA4404, selectable marker-free, *gus* homozygous lines were eventually obtained from 1.5 per 100 immature embryos inoculated. Compared to the segregation of uncoupled T-DNAs in conventionally produced progeny, the incorporation of haploid technology improves the time and resource efficiency of producing true-breeding, selectable marker-free transgenic barley.

**Electronic supplementary material:**

The online version of this article (doi:10.1007/s11103-012-9988-9) contains supplementary material, which is available to authorized users.

## Introduction

Barley is one of our major arable crop species, and may well have been the first species to have been domesticated. Among the temperate cereals it is one of the best adapted to low rainfall and poor soil conditions. Its grain is used both for animal feed and malting, with minor usage in the health food and bioethanol sectors. The simple genetics displayed by barley has for many years encouraged its exploitation as a genetic model, and more recently this has been extended into the field of transgenesis. Biolistic transformation was the earliest platform employed for this purpose (Wan and Lemaux 1994), but this has been largely replaced by *Agrobacterium*-mediated gene transfer, which can be applied to either immature embryos (Tingay et al. 1997), embryogenic pollen (Kumlehn et al. 2006) or isolated ovules (Holme et al. 2006). Although selectable markers are desirable for the efficient recovery of transgenic regenerants, they often have no further purpose once a transgenic plant has been developed. Moreover, the presence of a selectable marker prevents the use of the same gene for any successive round of transformation using another effector gene. In addition, retaining selectable markers which encode resistances to antibiotics is considered in some quarters to be somehow risky, and so commercially grown transgenic plants are often required to be free of those markers.

Several strategies have been elaborated to remove selectable markers from transgenic plants, while retaining the gene of interest (GOI). A particularly elegant one relies on site-specific recombination, in which the transformation cassette comprises, in addition to the GOI, the selectable marker flanked by specific recombinase recognition sites. The action of the relevant recombinase post transformation excises the selectable marker, leaving the GOI in situ (Dale and Ow 1991; Gleave et al. 1999; Kilby et al. 1995). The system based on the *Streptomyces* phage phiC31 integrase (Thorpe and Smith 1998) has been successfully applied for the elimination of selectable markers from transgenic plants and is particularly attractive, given its irreversibility. A second strategy relies on the activity of transposon systems; here, the selectable marker gene is flanked by sequences recognized by a transposase, so that when the cassette is introduced, the selectable marker is mobilized to a new location in the genome, thereby becoming separated from the GOI (Belzile et al. 1989; Cotsaftis et al. 2002; Gorbunova and Levy 2000). A third strategy involves the introduction of the selectable marker and the GOI on separate T-DNA sequences, and relies on their integration sites being different. Selectable marker-free plants retaining the GOI can then be selected, provided that the two transgenes are not linked in *cis* (McKnight et al. 1987). A co-transformation experiment of this type in rice and tomato has been reported by Komari et al. (1996), in which two T-DNAs were carried by a single plasmid, but separated from one another by some 15 kb. Its outcome was that over half of the regenerants were selectable marker-free but GOI positive. A similar experiment in barley, involving two T-DNAs separated from one another by only a short spacer, produced a co-transformation frequency of 66 %; the GOI was separable from the selectable marker in the progeny of about a quarter of the co-transformants (Matthews et al. 2001). Finally, some attempts have been made to avoid the use of selectable markers altogether (Holme et al. 2006). The various strategies to produce marker-free transgenic lines were extensively reviewed and discussed elsewhere (Hohn et al. 2001).

**Fig. 1:**
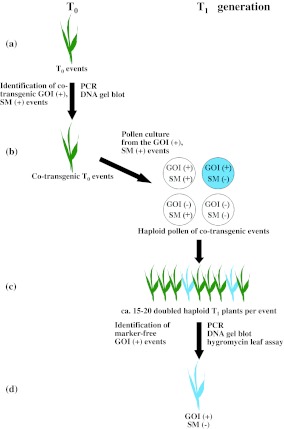
Schema for the production of selectable marker-free transgenic barley. **a** Immature embryos were used as the explant subjected to inoculation with *Agrobacterium*. **b** The selectable marker (SM, *hpt::gfp*) and the model gene-of-interest (GOI, *gus*) were co-transformed using separate T-DNAs. **c** Homozygous selectable marker-free GOI positive doubled haploid barley plants were regenerated from embryogenic pollen cultures. **d** If the two T-DNAs are inserted in different chromosomal locations, selectable marker-free GOI positive derivatives can be identified within the doubled haploid progeny

Here, we demonstrate that haploid technology that is widely used in barley breeding programmes can also be exploited for the efficient production of selectable marker-free transgenic barley plants. Our goal was to elaborate an effective transformation protocol based on immature embryo explants to deliver selectable marker-free, homozygous transgenic barley plants. The strategy selected was co-transformation of a selectable marker gene and the GOI, followed by their meiotic separation among doubled haploid progeny of the primary co-transformants. The virtue of this approach enables the rapid and efficient fixation of the GOI in selectable marker-free lines (Fig. 1).

## Materials and methods

### Construction of binary transformation vectors

**Fig. 2:**
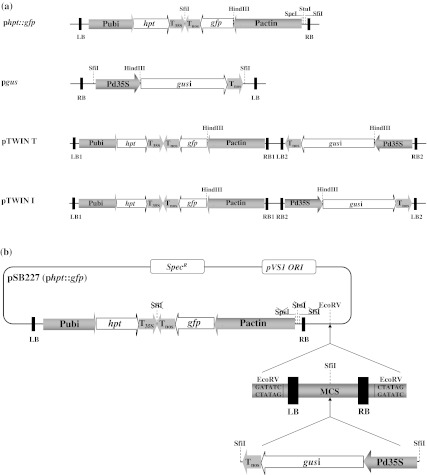
T-DNAs in a total of four constructs were used for *Agrobacterium*-mediated co-transformation of barley: **a** *hpt::gfp* was the selectable marker (SM), and *gus* the model gene-of-interest (GOI), the pTwin constructs contained both the SM and the GOI within the same vector in different orientations to one another. **b** the Twin binary vector pair containing two T-DNAs (*gus* and *hpt::gfp*) within a single plasmid. Pubi: maize *ubiquitin1* promoter; Pd35S: doubled enhanced CaMV *35S* promoter; Pactin, rice *actin* promoter; Tnos: *nopaline synthase* gene terminator; T35S: CaMV *35S* gene terminator; *gfp*: green fluorescent protein coding region; *gus*i: β-glucuronidase (*gusA*) protein coding region; *hpt*: hygromycin B phosphotransferase protein coding region; LB: left border; RB: right border; MCS: multicloning site; pVS1 ORI: *E. coli* origin of replication; *SpecR*: coding region for adenylyltransferase conferring bacterial resistance to spectinomycin

The binary vectors used were p6U (DNA Cloning Service, Hamburg, Germany) based plasmids, constructed using standard DNA cloning methods in the *E. coli* strains DH5α and DH10B (Sambrook et al. 1989). The pSB227 plasmid (designated later as p*hpt::gfp* to highlight its relevant elements) incorporates hygromycin phosphotransferase (*hpt*) as a selectable marker gene driven by the maize *ubiquitin1* promoter, fused to the *gfp*S65T coding sequence (Chiu et al. 1996) driven by the rice *actin1* promoter (McElroy et al. 1990) (Fig. 2a). The second binary vector, p*gus*, was obtained by replacing the *hpt* expression cassette in p6U with the *E. coli* ß-glucuronidase gene (*gus*) including the *StLS1* intron (Vancanneyt et al. 1990) driven by the cauliflower mosaic virus (CaMV) doubled enhanced *35S* (d35S) promoter (Odell et al. 1985). The Twin binary vectors harbour both T-DNAs separated by left and right border sequences (Fig. 2b). These vectors were generated by modifying pSB227 (p*hpt::gfp*) via digestion with *Spe*I and *Stu*I, followed by a 5–3′ exonuclease treatment and religation. This step also eliminated the *Sfi*I restriction site adjacent to the rice *actin1* promoter sequence, because it overlaps with the *Stu*I site. The second *Sfi*I restricion site between the *35S* and *nos* terminator sequences was then removed by *Sfi*I digestion, followed by a 3–5′ exonuclease treatment and religation. The Left Border-Multicloning Site-Right Border (LB-MCS-RB) fragment was PCR amplified by primers which incorporated flanking *Eco*RV restriction sites (5′-TAGATATCTGCAAGCTCCACCGGGTGCAAAGCGGCAGC and 5′-CCGATA TCATATCCGATTATTCTAATAAACGCTC) using the *hpt*-free p6U vector as template. The LB-MCS-RB fragment was then inserted into the modified pSB227 plasmid at the *Eco*RV site with the help of a TOPO-Cloning kit (Invitrogen) in both possible orientations. The *d35S::gus* sequence was released from the *hpt*-free p6U vector containing the d*35S*::*gus*i::T*nos* cassette by restriction with *Sfi*I and inserted into the pSB227 vector containing the multiple cloning site fragment flanked by the border sequences. This resulted in the two binary vectors pTwin T and pTwin I (Fig. 2a), differing in their orientation of *gus* in relation to *gfp*, with T standing for tandem and I for inverted.

### Barley genetic transformation

The transformation protocol applied to immature embryos and the generation of primary transgenic plants followed that of Hensel and Kumlehn (2004). Two *Agrobacterium tumefaciens* strains were used: a hypervirulent derivative of LBA4404 (Komari et al. 1996) and AGL-1 (Lazo et al. 1991). Genetic transformation of barley (*Hordeum vulgare* L.) was carried out using 14 different *Agrobacterium*/vector combinations, which are specified in Table 1. Each of the three replicates making up the entire experiment consisted of the inoculation of 90 immature embryos of the cultivar ‘Golden Promise’ with each of the 14 combinations. Because it was technically impossible to compare all 14 combinations in a single experimental run, combination 7 (a 1:1 mixture of LBA4404/p*hpt*:*gfp* and AGL-1/p*gus*) was included as an internal control in each transformation experiment. This ‘control’ was thus applied in a total of six replicates using 270 embryos each.

**Table 1:**
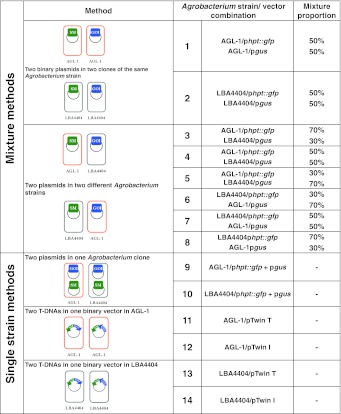
The 14 *Agrobacterium/*vector combinations used for co-transformation, involving two *Agrobacterium* strains (AGL-1 and LBA4404) and plasmids containing either *hpt::gfp*, *gus* or both

### Induction of embryogenic pollen cultures from primary co-transformants

Microspores at the highly vacuolated, pre-mitotic stage were isolated following Coronado et al. (2005). Other experimental details are as given by Kumlehn et al. (2006). Haploid regenerants were diploidized by subjection to colchicine treatment following Thiebaut and Kasha (1978).

### Isolation of plant DNA and PCR

**Fig. 3:**
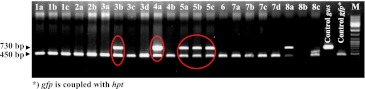
PCR analysis of primary transgenic (T_0_) plants. *Different numbers* indicate that regeneration occurred from different explants, while the *letters* are used to distinguish regenerants derived from the same explant. The co-transformants are *circled in red*. *M* DNA ladder

Genomic DNA of presumptive transgenic regenerants was used to establish the presence of both *gus* and *hpt::gfp* (Fig. 3). Fresh leaf material was homogenized in a mixer mill (Retsch MM301, Haan, Germany) and DNA was isolated following Palotta et al. (2000). PCRs were based on primer pairs specific for either *gfp* (5′-GGTCACGAACTCCAGCAGGA, 5′-GACCACATGAAGCAGCACGA) or *gus* (5′-CCGGTTCGTTGGCAATACTC, 5′-CGCAGCGTAATGCTCTACAC). Each PCR involved an initial denaturation step (95 °C/5 min), followed by 35 cycles of 95 °C/30 s, 60 °C/45 s, 72 °C/75 s, and ending with a final extension step (72 °C/7 min). PCR products were separated by electrophoresis through 1.2 % agarose gels. The length of the *gus* amplicon was 730 bp, and that of the *gfp* amplicon was 450 bp.

### DNA gel blot

The *gfp* and *gus* probes for the DNA gel blot were generated using the same primers as for the PCR. The amplicons were labelled with digoxygenin (PCR DIG Probe Synthesis Kit, Roche Diagnostics, Mannheim, Germany) for use as hybridization probes. Genomic DNA was digested with *Hin*dIII, separated by electrophoresis through an 0.8 % agarose gel, and transferred to a positively charged nylon membrane (Roche Diagnostics), following the manufacturer’s instructions. Each blot was hybridized first with the *gus* probe, then ‘stripped’ and reprobed with the *gfp* sequence. Hybridization, signal detection and probe stripping were carried out following the DIG Application Guide for Filter Hybridization Manual (Roche Diagnostics, Mannheim, Germany).

### Ploidy level analysis

The ploidy level of primary transgenic plants was determined using a flow cytometer (Ploidy Analyser 1, Partec, Münster, Germany) according to the manufacturer’s instructions.

### Histochemical detection of *gus* expression

GUS histochemical staining (Jefferson 1987) was applied to embryogenic callus and leaf tissue. The plant material was vacuum-infiltrated (ILMVAC, Laboratory Vacuum System, LVS 301 Zp, Ilmenau, Germany), then held overnight at 37 °C in 100 mM sodium phosphate buffer (pH 7.0) containing 0.1 % v/v Triton X-100, 10 mM EDTA, 1 mM X-gluc and 1.4 mM potassium ferricyanide. For leaf material, chlorophyll was first removed by treatment in 96 % ethanol at 60 °C for 2 h.

### Detection of *gfp* expression

Expression of *gfp* was screened in callus tissue and root tips, using a Leica MZFLIII fluorescence microscope equipped with the filter set GFP Plant (Leica Microsystems, Wetzlar, Germany).

### Leaf assay for hygromycin resistance

The rapid *hpt* assay described by Wang and Waterhouse (1997) was applied on leaf material harvested from plants using RM medium (Hensel and Kumlehn 2004) containing 200 mg/L hygromycin B. Leaves of plants free of *hpt* bleached on selective medium, while *hpt*-transgenics stay green over 1 week.

### Statistical treatment

Data were subjected to a parameter-independent Kruskal–Wallis one way analysis of variance on ranks (SigmaStat 3.0, SPSS Inc., Chicago, IL, USA). Pairwise comparisons of the *Agrobacterium*/vector combinations were performed against the respective control replicates that were conducted in the same experimental runs. *P* values < 0.05 were considered to indicate statistical significance.

## Results

### Generation of co-transformed T_0_ barley plants

**Fig. 4:**
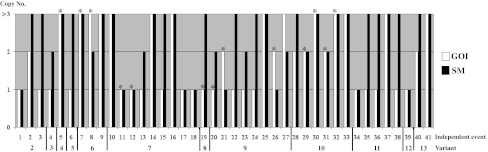
T-DNA copy numbers in independent co-transformants, and their segregation in the doubled haploid T_1_ generation as determined by DNA gel blot analysis. *T_1_ families which included segregants carrying the gene-of-interest (*gus*) but no selectable marker (*hpt::gfp)*

Four binary vectors were used (Fig. 2a), containing either *hpt::gfp* (the selectable marker) and/or *gus* (the GOI). In all, 14 combinations of *Agrobacterium* strain and vector were tested (Table 1). First, regenerants were tested by PCR. Plant genomic DNA containing only the *hpt* produced a single 450 bp band, while co-transgenics resulted an additional 730 bp amplicon representing the GOI *gus* (Fig. 3). A set of 606 regenerants carrying the selectable marker was derived from 5,130 inoculated embryos (Supplementary Table S1); these reflected the production of between one and 15 putative transgenic plants from each of 206 embryos. The sister plants derived from one single callus might either be genetically identical (clones) or represent independent lines. Of the regenerants, 129 (derived from 50 embryos) also carried *gus* (Supplementary Table S1). The stable integration of *hpt::gfp* and *gus* and their copy number was analysed by DNA gel blot, a procedure which was also able to recognize clonality among sister regenerants (Fig. 1a; Supplementary Table S1). About 30 % of these families included non-identical transformants, so that in total, 55 independent co-transformed events were obtained out of 228 independent transgenic events (Table 2, panel A and B; Fig. 1b). Of the 41 independent co-transgenic plants analysed by DNA gel blot, the GOI *gus* was present as a single copy in 48.8 %, as two copies in 17.1 %, and as three or more copies in 34.1 %. The equivalent frequencies for the selectable marker *hpt::gfp* were 22.0, 19.5 and 58.5 % as can be deduced from Fig. 4. The transformation efficiency (number of independent *hpt::gfp* positive plants per hundred inoculated embryos) of the various strain/vector combinations ranged from 0.7 to 9.6 %, with the most efficient combination (4) being a 1:1 mixture of AGL-1/p*hpt::gfp* and LBA4404/p*gus* (Table 2, panel A). There were statistically significant differences (*P* < 0.05) between the control (combination 7, see experimental procedures) and combinations 2, 9, 10 and 11 (Table 2, panel Transformation efficiency). The highest co-transformation efficiency (*hpt::gfp* positive plants carrying an additional *gus* per hundred inoculated embryos) was achieved from combination 9 (two plasmids in a single *Agrobacterium* clone), for which eight out of the 270 explants gave rise to independent co-transgenic lines. Six plants carrying both *gus* and *hpt::gfp* were regenerated from combinations 10 and 11 each, but a statistically significant difference with respect to the control could only be established for the latter (Table 2, panel B and Co-transformation efficiency).

**Table 2:** Summary of results obtained from the 14 *Agrobacterium*/vector combinations tested

Method	*Agrobacterium* strain/vector combination	A	Transformation efficiency (%)	B	Co-transformation efficiency (%)	C	D	GOI (+), SM-free production efficiency (%)
Two binary plasmids in two clones of the same *Agrobacterium* strain	1	11	4.1	2	0.7	1	0	0.0
	2	16	5.9*	5	1.9	3	0	0.0
	7 (replicates run with 1, 2)	5	1.9	3	1.1	3	1	0.4
Two plasmids in two different *Agrobacterium* strains	3	21	7.8	2	0.7	1	0	0.0
	4	26	9.6	2	0.7	2	2	0.7
	5	4	1.5	1	0.4	1	0	0.0
	7 (replicates run with 3, 4, 5)	15	5.6	4	1.5	4	1	0.4
Two plasmids in two different *Agrobacterium* strains	6	9	3.3	3	1.1	3	2	0.7
	7 (replicates run with 6, 8)	13	4.8	4	1.5	4	0	0.0
	8	13	4.8	1	0.4	1	1	0.4
Two plasmids in one *Agrobacterium* clone	9	24	8.9*	8	3.0	6	3	1.1
	10	15	5.6*	6	2.2	4	4	1.5*
	7 (replicates run with 9, 10)	5	1.9	2	0.7	2	0	0.0
Two T-DNAs in one binary vector in AGL-1	11	15	5.6*	6	2.2*	4	0	0.0
	12	6	2.2	1	0.4	1	0	0.0
	7 (replicates run with 11, 12)	2	0.7	0	0.0	0	0	0.0
Two T-DNAs in one binary vector in LBA4404	13	10	3.7	2	0.7	1	0	0.0
	14	6	2.2	2	0.7	1	0	0.0
	7 (replicates run with 13, 14)	12	4.4	1	0.4	1	0	0.0
	7 (sum of controls)	52	3.2	14	0.9	14	2	0.1

*A* number of independent primary transgenic (*hpt::gfp* positive) plants; *B* number of independent co-transgenic (*hpt::gfp* and *gus*-positive) plants; *C* number of independent co-transgenic plants producing green doubled haploid progeny; *D* number of independent co-transgenic plants producing GOI-positive, selectable marker-free green doubled haploid progeny

* Higher efficiency of the *Agrobacterium*/vector combination on a statistically significant level (*P* < 0.05) as compared to the control repetition conducted in the same experimental run

### Doubled haploids bred from co-transformant selections

**Fig. 5:**
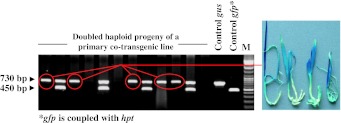
Identification of selectable marker-free, *gus* homozygous individuals. Multiplex PCR analysis tracking the segregation of *gus* and *hpt*::*gfp* in a family of doubled haploid T_1_ plants (*left*). Expression of *gus* in marker-free doubled haploid seedlings, as demonstrated by histochemical analysis (*right*). *M* DNA ladder

Embryogenic pollen cultures were induced from the immature spikes of 43 of the 55 co-transformants (Table 2, panel B and C). The mean frequency of green doubled haploid regenerants from these cultures was 1.4 per spike (varying from 0.1 to 4.3, data not shown). This rate was sufficient to produce around 15 doubled haploid progeny per plant, as each produced an average of 10.7 harvestable spikes. Three assays (PCR, hygromycin leaf assay and DNA gel blot) were applied to determine whether *gus* segregated independently from the selectable marker among the doubled haploid progeny (Figs. 1c, 4, 5). First, those plants were selected by PCR which produced a single 730 bp *gus* band but lacked the selectable marker, and GUS staining was carried out (Figs. 1c, 5). Hygromycin leaf assay corroborated the PCR results, as leaves of Hygromycin-sensitive plants bleached on medium containing a high level of hygromycin. The absence of linkage was detected in 31 of the 43 doubled haploid families. However, because some of the transformants carried at least one copy of both transgenes linked to one another, the number of T_1_ DH families in which *gus* could be separated from *hpt::gfp* was just 14 (Table 2, panel D; Fig. 1d). The DNA gel blot profiles were informative with respect to both transgene copy number and also linkage between the transgenes. Three of the eight combination 9 transformants gave rise to selectable marker-free *gus* positive doubled haploid progeny (equivalent to 1.1 lines per 100 embryos), while combination 10 produced an efficiency of 1.5 lines per 100 inoculated embryos. Selectable marker-free *gus* positive doubled haploids were also produced from combinations 4, 6, 7 and 8, but not from combinations 1, 2, 3, 5, 11, 12, 13 or 14 (Fig. 4).

One of the combination 8 transformants produced exclusively selectable marker-free *gus* positive progeny, even though it was known from DNA gel blot analysis that the transformation event involved five copies of the *hpt*::*gfp* transgene (data not shown). This indicates that the primary transgenic plant was chimeric with regard to the *hpt::gfp* insertion locus at which all of these five copies were likely to be linked, and that *hpt::gfp*-positive tissue of this chimera has not been involved in the formation of the spikes used to generate DH-lines.

The Twin (T and I) *Agrobacterium/*vector combinations (11–14) derived co-transformants did not result in any marker-free *gus* positive DH line (Table 2, panel D). Note, however, that it was possible to recover progeny from co-transformants derived from combinations 11 and 13 which carried *hpt::gfp* but not *gus*, showing that the two T-DNAs can be separately inserted from a Twin vector (data not shown). In these transformants, it appears that multiple (three or more) copies of *hpt::gfp* had been inserted, with one or more of these insertion sites also containing a copy of *gus*. According to DNA gel blot analysis of DH progeny, one derivative of combination 13 included a multi-event involving the integration of eight *hpt::gfp* T-DNA copies at five separate loci.

### Ploidy variation among the primary transgenic regenerants

Spontaneous genome doubling can occur when plants are regenerated from embryogenic cultures (Bregitzer et al. 1998; Choi et al. 2000; Gaponenko et al. 1988). A number of the primary transgenic regenerants developed into plants which were abnormally tall, produced long, wide leaves and flowered late, and the application of flow cytometry demonstrated that five of these were indeed tetraploid. Among the progeny of two of these five plants, *gus* segregated independently of the selectable marker. Flow cytometry analysis also showed that the progeny regenerated from embryogenic pollen culture of one of these plants included 18 diploid and two tetraploid individuals, while the other produced eight diploids and six tetraploids.

### Time frame

**Fig. 6:**
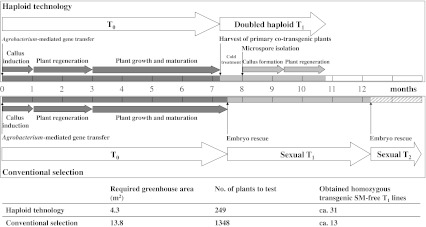
Unequal efforts are required for the production and identification of selectable marker-free, GOI homozygous transgenic lines employing haploid technology as compared to conventional segregation in the most efficient transformation method ‘two plasmids in one *Agrobacterium* clone’ (combinations 9 and 10)

Figure 6 presents a time line for the production of doubled haploid transgenic barley selections, achieved using *Agrobacterium*-mediated co-transformation followed by immature pollen culture-based generation of doubled haploid progeny in the most efficient method ‘two plasmids in one *Agrobacterium* clone’ (combinations 9 and 10). This method generated a total number of 7 independent lines producing selectable marker-free doubled haploid progeny containing the GOI (Table 2, panel D). In order to select such individuals, 249 microspore culture-derived T_1_ DH plants were analysed for the presence/absence of the two T-DNAs (data not shown). In a presumptive conventional selection of plants from sexual T_2_ populations, analysis of 1,348 individuals would be required to obtain just 13 desired homozygous *gus*-positive lines lacking *hpt*. The whole process exercised in the present study took about 43 weeks to move from the dissection of immature embryos to the identification of homozygous transgenic, selectable marker-free plants, a saving of at least 13 weeks and significant effort in genotype analysis over the conventional process based on segregation in selfing generations.

## Discussion

### Transformation efficiency

Various gene transfer protocols have been devised to obtain co-transformation—these include the use of two bacterial strains (Daley et al. 1998; De Framond et al. 1986), and a single strain carrying either two independent plasmids (Daley et al. 1998; De Framond et al. 1986; Komari et al. 1996) or a single plasmid containing two T-DNAs (Komari et al. 1996; Stahl et al. 2002). Successful co-transformation requires the induction of sufficient independent events to allow for the separation of the selectable marker from the GOI via conventional segregation. Here, we present experiments in which a range of co-transformation strategies were compared, involving either two *Agrobacterium* clones, two plasmids within a single clone, or a clone harbouring a plasmid carrying two T-DNAs. The *hpt*::*gfp* transformation efficiencies achieved in these experiments ranged from 0.7 to 9.6 % (Table 2), rates which are comparable with current protocols based on immature embryo explants of cv. “Golden Promise” (Goedeke et al. 2007; Hensel and Kumlehn 2009). Transgenics carrying either the selectable marker only or both transgenes were recovered from each strain/vector combination. The overall transformation efficiency was not correlated with the co-transformation efficiency, and only combination 11 (AGL-1/pTwin T) differed significantly from its control combination for both these aspects (Table 2). Combinations 3 and 4 (both representing the ‘two plasmids in different *Agrobacterium* strains’ situation) were effective with respect to the rate of formation of primary transgenic events. However, the rate of recovery of co-transformation events was rather poor. The lack of association between genetic transformation and co-transformation of barley cv. ‘Golden Promise’ is probably due the fact, that presence of the second T-DNA containing the gene-of-interest does not provide the plants with any benefit in the regeneration process.

Although segregation of the two T-DNAs was observed in more than half of the doubled haploid progenies, *gus* positive plants lacking the selectable marker were only recovered from a half of these (data not shown). Only six of the 14 strain/vector combinations gave rise to the desired class of progeny, and neither the ‘two plasmids present in two clones of the same strain’ method nor the Twin method produced any selectable marker-free doubled haploids containing the GOI. The most effective method involved the presence of both plasmids in a single bacterial clone, irrespective of the identity of the *Agrobacterium* strain. However, the recovery rate of selectable marker-free *gus* positive doubled haploid progeny per 100 inoculated explants was significantly greater than that achieved in the control combination only for LBA4404 (Table 2, panel GOI (+), SM-free production efficiency). Using this combination (10), selectable marker-free *gus* positive doubled haploids were produced from 1.5 out of 100 explants, whereas this recovery rate was just as high as 0.7 in the ‘two plasmids in two different strains’ method (combinations 4 and 6).

Following transgenesis, haploid technology provides a means of generating selectable marker-free plants homozygous for the GOI without the need for further selfing generations. A comparison for the most efficient method ‘two plasmids in one *Agrobacterium* clone’ (combinations 9 and 10) between the recovery of selectable marker-free, *gus* positive individuals using haploid technology and the expected outcome of the same transgenic situation based on selection for homozygosity in the sexual T_2_ generation is given in Fig. 6. This method generated 39 independent primary transgenic plants, of which 14 involved co-transformation (Table 2, panel A and B). Of these, doubled haploid progeny lacking the selectable marker but containing the GOI were produced from seven plants (Table 2, panel D). A further advantage of applying haploid technology is a significant reduction in space and effort required, since the conventional method requires the testing of larger numbers of T_2_ individuals to detect selectable marker-free lines which are also homozygous for the GOI. In comparison, in sexual T_1_ populations with Mendelian segregation of unlinked T-DNAs, most of the plants testing positive for the GOI and lacking the selectable marker are expected to be hemizygous for the transgene, which requires a follow-up selection of homozygous lines that are ultimately needed for breeding purposes.

### Integration of recombinant DNA in the barley genome

The literature suggests that typically, the process of *Agrobacterium*-mediated DNA-transfer applied to immature barley embryos produces between one and three T-DNA inserts per event, with only around 10 % of events involving four or more insertions (Hensel et al. 2008; Lange et al. 2006; Travella et al. 2005). About one half of all multiple inserts involve tandem or head-to-head insertions at a single site (Stahl et al. 2002). Across the analysed set of 41 regenerants in the present experiments, the *gus* transgene copy number was from one to two in ca. 66 % of the primary co-transgenic individuals, although the average copy number of the *hpt::gfp* sequence was rather higher (Fig. 4). The T-DNA copy number itself does not give any information about the number of integration loci, e.g. a high number of transgene copies is not necessarily associated with many integration loci. Pursuing the pattern of transgene segregation in the DH T_1_ populations is more conclusive. This can indicate if there are loci where more than one transgene copy was integrated in the genomic DNA of the plant cell by the gene transfer apparatus of *Agrobacterium*. In fact, multiple T-DNAs were often integrated linked to each other in the plant genome and behaved like a single locus (Hensel et al. 2008). In the present study, such linkage groups were frequently found among the combinations (data not shown). The failure to recover selectable marker-free *gus* positive segregants from the Twin vectors generated co-transformants suggests that these vectors favour the integration of the two T-DNAs at a single site, possibly owing to frequent misinterpretation of the two adjacent T-DNAs as a single one by the gene transfer machinery of *Agrobacterium*. It is possible that increasing the length of the spacer sequence separating the two T-DNAs (in the present experiments this was just 500 bp) may have improved the chances of obtaining independent insertions, as suggested by Matthews et al. (2001).

De Block and Debrouwer (1991) have suggested that the identity of the *Agrobacterium* strain used can influence the pattern of T-DNA insertion; specifically, nopaline-derived strains such as AGL-1 tend to favour linked co-insertions, while octopine-derived ones such as LBA4404 tend to favour unlinked ones. Our data suggest that the pattern of T-DNA integration is dependent on both the construct and the *Agrobacterium* strain. Using both strains or the ‘two plasmids in one clone’ method, the independent insertion of *gus* was a relatively frequent event. In contrast, the Twin combinations tended to favour high *hpt::gfp* transgene copy numbers.

### Further characteristics of gene transfer and DH production

Plants regenerated from the same callus may well not be clonal, as shown for example in rice by Sallaud et al. (2003). DNA gel blot-based profiling of the present materials identified both copy number and fragment size variation between those ‘sister’ regenerants (data not shown). In the present study, some 2 % of the inoculated embryos produced more than one transgenic regenerant. The analysis of 101 regenerants derived from 20 of those embryos revealed that 6 of them had given rise to more than one genetically independent transgenic line, i.e. a total of only 28 out of the 101 transgenic regenerants proved to be independent. Consequently, a sensible routine practice would be to retain only one regenerant per explant.

Somaclonal variation occurs due to transformation and tissue culture processes (Bregitzer et al. 1998; Choi et al. 2001; Lemaux et al. 1999). Barley genetic transformation based on biolistics frequently produces tetraploid regenerants, which look abnormal and are partially sterile. At least 10 % of the primary co-transgenic regenerants obtained in the present study proved to be tetraploid (data not shown). They included some which generated *gus* positive, hygromycin-sensitive progeny. Microspore isolation from the spikes of such tetraploid barley plants was possible, and successful embryogenic pollen cultures producing green regenerants were obtained. Embryogenic pollen culture-derived progeny from the tetraploids were diploid (dihaploid), but not necessarily homozygous. Tetraploid (doubled ‘dihaploid‘) plants were obtained as well, following spontaneous genome doubling. These regenerants were successfully tested in a successive round of transformation in order to assess their potentially improved amenability for *Agrobacterium*-mediated gene transfer (data not shown).

## Electronic supplementary material

Below is the link to the electronic supplementary material.

Table S1. Overview of plants produced in the course of the co-transformation experiments. (PPT 373 kb)
