# Supplementary material for: The elimination of a selectable marker gene in the doubled haploid progeny of co-transformed barley plants
Source: Plant Mol Biol. 2012 Nov 21;81(1):149–60. doi: 10.1007/s11103-012-9988-9 (PMC3527739; doi:10.1007/s11103-012-9988-9)
Supplement: Supplementary file 1 — Table S1. Overview of plants produced in the course of the co-transformation experiments. (PPT 373 kb) [file 11103_2012_9988_MOESM1_ESM.ppt]

## Slide 1
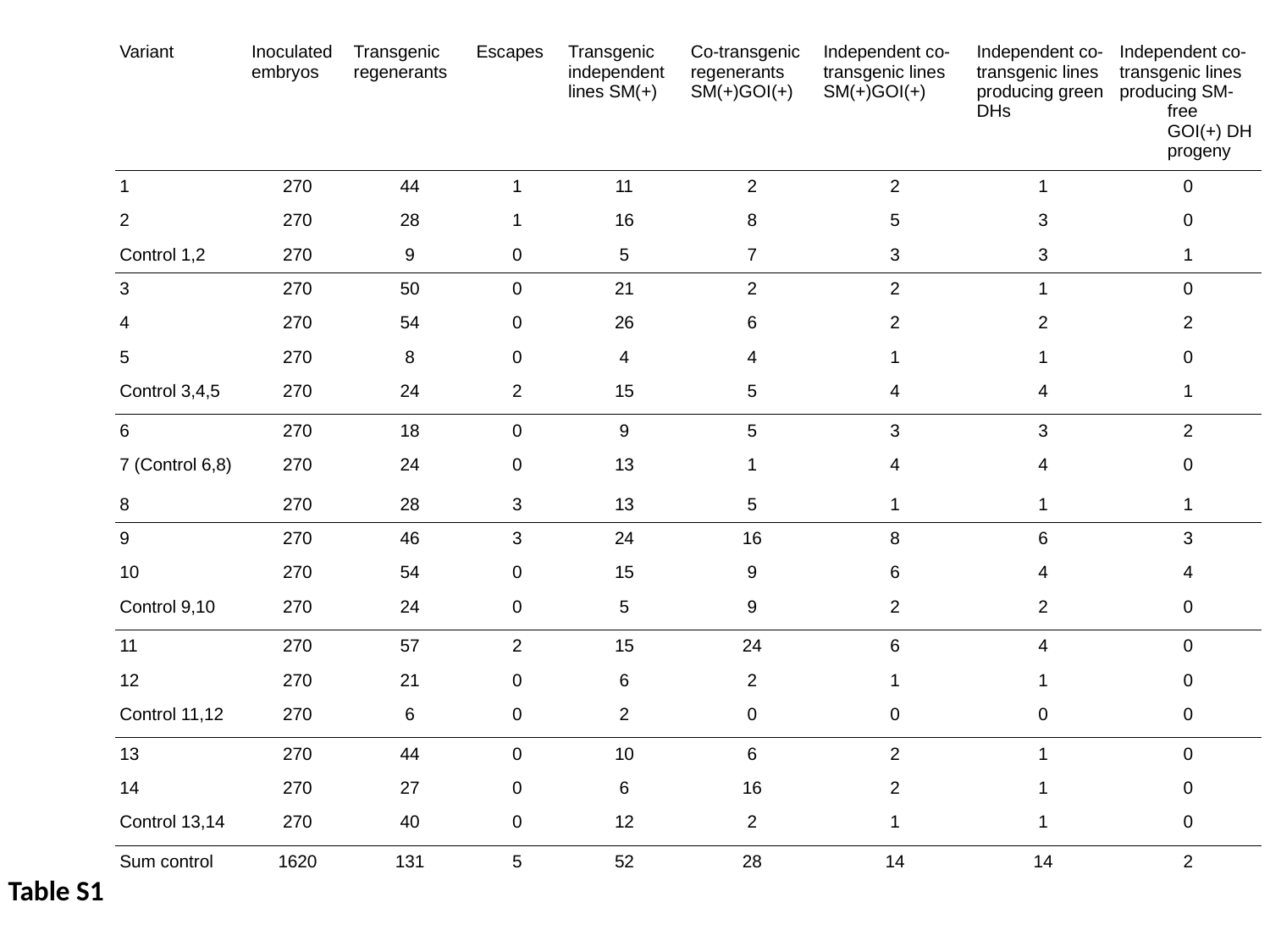

| Variant | Inoculated embryos | Transgenic regenerants | Escapes | Transgenic independent lines SM(+) | Co-transgenic regenerants SM(+)GOI(+) | Independent co- transgenic lines SM(+)GOI(+) | Independent co- transgenic lines producing green DHs | Independent co- transgenic lines producing SM-free GOI(+) DH progeny |
| --- | --- | --- | --- | --- | --- | --- | --- | --- |
| 1 | 270 | 44 | 1 | 11 | 2 | 2 | 1 | 0 |
| 2 | 270 | 28 | 1 | 16 | 8 | 5 | 3 | 0 |
| Control 1,2 | 270 | 9 | 0 | 5 | 7 | 3 | 3 | 1 |
| 3 | 270 | 50 | 0 | 21 | 2 | 2 | 1 | 0 |
| 4 | 270 | 54 | 0 | 26 | 6 | 2 | 2 | 2 |
| 5 | 270 | 8 | 0 | 4 | 4 | 1 | 1 | 0 |
| Control 3,4,5 | 270 | 24 | 2 | 15 | 5 | 4 | 4 | 1 |
| 6 | 270 | 18 | 0 | 9 | 5 | 3 | 3 | 2 |
| 7 (Control 6,8) | 270 | 24 | 0 | 13 | 1 | 4 | 4 | 0 |
| 8 | 270 | 28 | 3 | 13 | 5 | 1 | 1 | 1 |
| 9 | 270 | 46 | 3 | 24 | 16 | 8 | 6 | 3 |
| 10 | 270 | 54 | 0 | 15 | 9 | 6 | 4 | 4 |
| Control 9,10 | 270 | 24 | 0 | 5 | 9 | 2 | 2 | 0 |
| 11 | 270 | 57 | 2 | 15 | 24 | 6 | 4 | 0 |
| 12 | 270 | 21 | 0 | 6 | 2 | 1 | 1 | 0 |
| Control 11,12 | 270 | 6 | 0 | 2 | 0 | 0 | 0 | 0 |
| 13 | 270 | 44 | 0 | 10 | 6 | 2 | 1 | 0 |
| 14 | 270 | 27 | 0 | 6 | 16 | 2 | 1 | 0 |
| Control 13,14 | 270 | 40 | 0 | 12 | 2 | 1 | 1 | 0 |
| Sum control | 1620 | 131 | 5 | 52 | 28 | 14 | 14 | 2 |
Table S1
